# Evolutionary Game and Simulation of Green Housing Market Subject Behavior in China

**DOI:** 10.1155/2022/7153270

**Published:** 2022-04-05

**Authors:** Yingmiao Qian, Mengyuan Yu, Tao Wang, Ruijia Yuan, Zhenan Feng, Xing Zhao

**Affiliations:** ^1^School of Management Science and Engineering, Anhui University of Finance and Economics, Bengbu 233030, China; ^2^School of Management, Hefei University of Technology, Hefei 230009, China; ^3^School of Public Administration, Chongqing University, Chongqing 400030, China; ^4^School of Built Environment, Massey University, Palmerston North 4442, New Zealand

## Abstract

In China, driven by the national “3060” double carbon targets (i.e., reaching peak carbon emissions by 2030 and carbon neutrality by 2060), green housing has become one of the major fields to reduce carbon emissions, facilitating the achievement of the double carbon targets. Promoting the growth of green housing is an important way for the real estate industry to achieve low-carbon transformation and improve the quality of housing. Meanwhile, the construction industry also can benefit from green housing to achieve its energy conservation and emission reduction targets. Therefore, it is critical to boost and maintain the sustainable growth of the green housing market in China. However, the literature has not focused attention on the market behavior of the green housing market in China. This study proposes a tripartite evolutionary game model to investigate the subject behavior of the green housing market in China. This model consists of three major subjects in a green housing market: developers, consumers, and governments. Based on this model, this study analyzes the stability of the strategy options for each stakeholder and identifies the stable conditions of strategy portfolios to reach the equilibrium points of the game system. The validity of the proposed tripartite evolutionary game model is tested through the simulation of the impacts from various factors on system evolution. According to the impacts of factors and the stable conditions of strategies, this paper puts forward relevant policy suggestions for the healthy and sustainable growth of China's green housing market.

## 1. Introduction

Natural resources are being consumed enormously as a result of human uncontrolled exploitation, the resulting series of environmental problems such as greenhouse effect and extreme climate disasters has become increasingly serious [1]. The construction industry is an important area of final energy consumption and carbon dioxide emissions. According to the report released by the United Nations Environment Programme (UNEP), the construction industry accounted for 36% of the global final energy consumption and 37% of the energy-related carbon dioxide emissions in 2020 [2]. Therefore, energy conservation and emission reduction in the construction industry is crucial to curb global climate change and achieve carbon neutrality goals. The high consumption brought about by traditional buildings has a serious negative impact on the sustainable development of society. In order to reduce the environmental load and achieve the development strategy of buildings adapting to the ecological environment, the green building comes into being. Green buildings have the characteristics of energy conservation, environmental protection, and resource conservation, as well as high economic benefits [3–6], which have a broad market prospect in advocating sustainable development today. Green housing is an important branch of green building. It emphasizes the harmonious coexistence of residential area and environment. Therefore, the impact on the natural environment is taken into account in all activities such as design, construction, and operation, and the negative impact is controlled to the minimum as far as possible [7]. It can be said that green housing is the key to promote the high-quality development of housing construction and promote the green and sustainable development of cities. However, in the context of the country's vigorous development of green buildings, the market development of green housing still faces many problems. First, the low level of technological innovation and high development cost fundamentally hinder the development process [8, 9]; Second, for consumers, they often do not understand the benefits and health value of green housing but are unwilling to buy it because of its incremental cost [10]. Moreover, for developers, the development of green housing faces the dilemma of mismatch between income and expenditure, which hinders the promotion of green housing to a large extent [11]. Therefore, although green housing has the advantages of environment, society, and economy, they will only be considered for the development and adoption of green housing if the interests of major market players are guaranteed.

At this time, using incentive as a driving tool can encourage the adoption of green building and green housing to a certain extent [12, 13]. In general, incentives can be divided into external incentives and internal incentives. External incentives mainly come from the forced promotion of the government, which requires beneficiaries to meet specific conditions or requirements before benefiting, while internal incentives refer to attracting beneficiaries to be incentivized out of volition due to the value of green buildings [14]. Despite their differences, the vast majority of scholars recognize the positive role of the two. Guo et al. [15] affirmed the importance of government incentives and pointed out that legislation is the most effective way to promote the development of green buildings in Hong Kong, while expedited permits and density bonus can encourage market players to adopt green environmental protection voluntarily. He and Chen [16] found that the government subsidy policy has a positive incentive effect for the development of green buildings. Simultaneously subsidizing both developers and consumers obtain the highest social welfare. Therefore, in order to improve the popularity of green buildings, incentives should be provided to both developers and consumers. Bond [17] argued that the government sees the benefits of sustainable construction more through social and environmental benefits, while the private sector is driven more by economic returns. Moreover, when some investors pay more and more attention to corporate social responsibility and social responsibility investment, they will be more willing to promote the construction of green buildings. Love et al. [18] confirmed the catalytic role of clients in driving the sustainability agenda through a specific green building case, where building performance can be significantly improved when the client have a sustained drive and commitment to innovation.

The abovementioned research supports the effectiveness of green building incentive, but mainly focuses on specific incentive measures and policies. In the green building market, the key stakeholders have complex relationships and different needs; it is thus necessary to study the behavior strategies among them. Meng et al. [19] studied the behavior evolution process of the two main stakeholders of green building (contractors and government departments) under different reward and punishment policy combinations, so as to provide useful suggestions for the government to formulate reasonable incentive policies. Cohen et al. [20] used the prisoner's dilemma model to point out that developers and consumers do not adopt green houses, which belong to a suboptimal equilibrium. The government should provide incentives to developers and consumers, so as to make the green housing market move toward the optimal balance. Feng et al. [21] found the interest equilibrium point among green building stakeholders (government, construction units and consumers) by building a game model, so as to provide reference for the development of green building led by the government. Chen [22] analyzed the economic benefits of green building by building a bilateral game model between green building developers, consumers, and the government. The research found that the incremental profit of developers is the primary factor affecting enterprise decision-making, followed by the government's incentive policy, and the final strategic choice will be stabilized to higher economic benefits. Most literature analyzed the behavior strategies and interest conflicts of various subjects in the green building market, where the game relationship between government and developers; government and consumers; developers and consumers; government, developers, and consumers are key topics [23–25].

Throughout the existing literature, it is either limited to a single perspective, such as the incremental costs or payoffs of a tripartite subject and the equilibrium points of interests of subjects in a market, which fails to comprehensively look into the evolutionary path of the subject behavior of a green housing market or it focuses on how to formulate incentive policies to promote the growth of a green housing market; however, the benefits brought by incentive policies and consequently, the impacts of these benefits on the decision-making of governments are overlooked. For instance, how will a government make decisions and other subjects behave if incentive policies fail to deliver more benefits? Meanwhile, some researchers have built relevant models to analyze the behavioral evolution of green housing market subjects, but there is still little theoretical understanding of these models. Furthermore, the dynamic simulation and validation of these models are lacking.

Therefore, in order to bridge this knowledge gap, this study proposes a tripartite evolutionary game model, which considers developers, consumers, and governments, to investigate the subject behavior of the green housing market in China. This study also simulates the evolutionary path of the green housing market to validate the proposed model. The paper consists of the following parts: Section 1 introduces the background and summarizes the relevant literature.  Section 2 puts forward model assumption and establishes the game model.  Section 3 analyzes the model formulation.  Section 4 carries on the numerical simulation.  Section 5 draws the conclusion and implication. This study will provide some reference value to the healthy and sustainable development of the green housing market in China.

## 2. Model Assumption and Formulation

### 2.1. Model Assumption

It is well known that the development of green housing market involves multistage decision-making by multiple stakeholders, among which the developers^①^, consumers^②^, and government^③^ are the core subjects. The evolutionary game with them has the following five assumptions.

**Assumption 1 .**

In view of the fact that green housing market subjects are not completely rational and accurate in the process of acquiring knowledge and information, all three subjects are participants of bounded rationality who constantly adjust their strategies in the process of interaction and stabilize to optimal strategies over a period of evolution.

**Assumption 2 .**

The developers' strategic space  *α* = (*α*_1_, *α*_2_) = (developing green housing, developing ordinary housing); Developers have a probability of *x* to choose *α*_1_ and a probability of (1 − *x*) to choose *α*_2_. The consumers' strategic space *β* = (*β*_1_, *β*_2_) = (purchasing green housing, purchasing ordinary housing); Consumers have a probability of *y* to choose *β*_1_ and a probability of (1 − *y*) to choose *β*_2_. The governments' strategic space *γ* = (*γ*_1_, *γ*_2_) = (providing incentive policies, providing no incentive policies); Governments have a probability of *z* to select *γ*_1_ and a probability of 1 − *z* to select *γ*_2_. *x*, *y* and *z* all belong to [0, 1].

**Assumption 3 .**

*A*_*P*_ is the sales payoff of developers from ordinary housing, while *C*_*P*_ is the development cost of ordinary housing. Meanwhile, *A*_*P*_ is also the spending of consumers purchasing ordinary housing. In the case of green housing, the incremental sales payoff from green housing is *A*_*Z*_, the incremental development cost on green housing is *C*_*z*_, and the potential payoff brought by green housing for developers is *A*_*Q*_. The potential payoff has several streams, including the increased brand value, social image, and reputation resulted from developing green housing, the preferential treatments of taxation and mortgage, and the savings of land use and building materials in the process of development. In addition, when governments provide subsidies as incentive polices, developers get a subsidy of *θD*_2_ from developing green housing, where *θ* is the proportion of governments' subsidy *D*_2_.

**Assumption 4 .**

The payoffs of consumers is *S*_*P*_ when they purchase ordinary housing, with a spending of *A*_*P*_. When purchasing green housing, the incremental residential utility payoffs obtained by consumers is *φS*_*Z*1_, which includes the financial benefits from the savings of energy and water in the use of green housing. The incremental perception gains obtained by consumers is *ηS*_*Z*2_, which includes the satisfaction and comfort brought by green housing to consumers. The incremental spending paid by consumers on green housing is *A*_*Z*_. In addition, when governments provide subsidies as incentive polices, consumers who purchase green housing get subsidies (1 − *θ*) *D*_2_, where *θ* is the proportion of governments' subsidy *D*_2_ received by developers.

**Assumption 5 .**

Governments have two strategies: either providing incentive polices or not. In the case of providing incentive polices, a management cost *D*_1_ will occur as governments have to spend on the propaganda for environmental protection and the regulation and monitoring on environment to facilitate the social promotion of green housing. Consequently, the utility of the use of *D*_1_ is *G*_1_. Meanwhile, the social and environmental benefits brought by green housing are *G*_2_. When both developers and consumers choose green housing, the total amount of subsidies given by governments as incentive polices is *D*_2_. When developers choose green housing and consumers purchase ordinary housing, governments bear social environmental risk costs, which are *D*_3_.

### 2.2. Model Formulation

**Figure 1:**
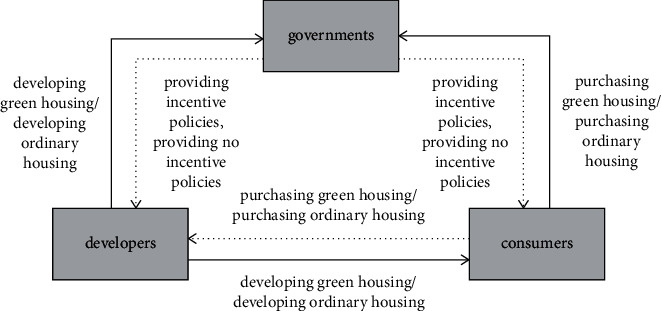
Logical relationship between each subject in the evolutionary game of a green housing market.

According to the previous literature [21–25], combining with the abovementioned relative assumption, the tripartite evolutionary game model of a green housing market has been constructed. Moreover, the logical relationship between each subject in the evolutionary game is shown in Figure 1. Whether or not government incentives, developers have two choices: to develop green housing or to develop ordinary housing; consumers similarly have two choices: to purchase green housing or to purchase ordinary housing, the strategies are marked with arrows. Meanwhile, the mixed strategy payoff matrix of developers, consumers, and governments is constructed with the assumptions stated in Section 2.1, as shown in Table 1.

## 3. Derivation and Analysis of the Model Formula

### 3.1. Replicator Dynamics Equations and Phase Diagrams of Each Game Subject's Decision-Making

#### 3.1.1. Developers

The expected payoff *E*_11_ of developers developing green housing, *E*_12_ of developing ordinary housing, and the average expected payoff *E*_1_ are given below:

(1) $$\begin{array}{c} \left\{\begin{array}{c} E_{11}=yz\left[\left(A_{p}+A_{Z}\right)-\left(C_{P}+C_{Z}\right)+\theta D_{2}+A_{Q}\right]+y\left(1-z\right)\left[\left(A_{p}+A_{Z}\right)-\left(C_{P}+C_{Z}\right)+A_{Q}\right]+\left(1-y\right)z\left[-\left(C_{P}+C_{Z}\right)+\theta D_{2}+A_{Q}\right]\\ +\left(1-y\right)\left(1-z\right)\left[-\left(C_{P}+C_{Z}\right)+A_{Q}\right],\\ E_{12}=yz\left(-C_{P}\right)+y\left(1-z\right)\left(-C_{P}\right)+\left(1-y\right)z\left(A_{P}-C_{P}\right)+\left(1-y\right)\left(1-z\right)\left(A_{P}-C_{P}\right),\\ E_{1}=xE_{11}+\left(1-x\right)E_{12}, \end{array}\right) \end{array}$$

The replicator dynamics equation of residential developers' decisions is as follows:

(2) $$\begin{array}{c} F\left(x\right)=\frac{\text{d}\left(x\right)}{\text{d}t}=x\left(E_{11}-E_{{}^{1}}\right)=x\left(1-x\right)\left[y\left(2A_{P}+A_{Z}\right)+z\theta D_{2}+A_{Q}-A_{P}-C_{Z}\right]. \end{array}$$

The first derivatives with respect to *x* and the set *G* (*y*) are, respectively, as shown below:

(3) $$\begin{array}{c} \frac{\text{d}\left(F\left(x\right)\right)}{\text{d}x}=\left(1-2x\right)\left[y\left(2A_{P}+A_{Z}\right)+z\theta D_{2}+A_{Q}-A_{P}-C_{Z}\right],\\ G\left(y\right)=\left[y\left(2A_{P}+A_{Z}\right)+z\theta D_{2}+A_{Q}-A_{P}-C_{Z}\right]. \end{array}$$

**Figure 2:**
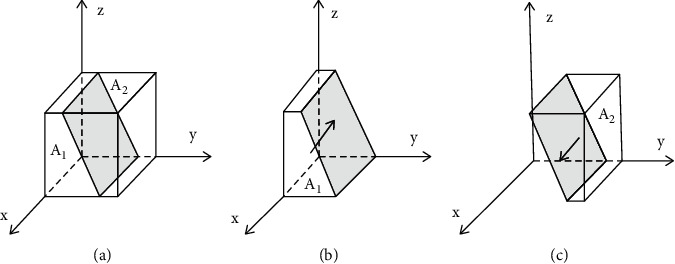
Evolution phase diagrams of developers' decisions in the case of develop green housing. (a) *y*=*y*^*∗*^, *y* is equal to the stable point. (b) *y* < *y*^*∗*^, *y* is less than the stable point. (c) *y* > *y*^*∗*^, *y* is greater than the stable point.

Based on the stability principle of differential equations, the probability of developers choosing developing green housing in a stable state must be satisfied by *F*(*x*)=0, where d(*F*(*x*))/d*x* < 0. Because ∂*G*(*y*)/∂*y* > 0, *G*(*y*) is the increasing function of *y*. Therefore, when *y*=[(*A*_*P*_+*C*_*Z*_) − *A*_*Q*_ − *zθD*_2_]/(2*A*_*P*_+*A*_*Z*_)=*y*^*∗*^, *G*(*y*)=0; at this point, d(*F*(*x*))/d*x* ≡ 0, *F*(*x*) ≡ 0, which means that in a group of developers, any proportion of individuals choosing developing green housing is a stable strategy. When *y* < *y*^*∗*^, *G*(*y*) < 0, *F*′(*x*)*|*_*x*=0_ < 0, *F*′(*x*)*|*_*x*=1_ > 0; at this point, *x* = 0 is the stable evolution point of developers, which means that when the proportion of consumers choosing purchasing green housing is less than [(*A*_*P*_+*C*_*Z*_) − *A*_*Q*_ − *zθD*_2_]/(2*A*_*P*_+*A*_*Z*_), developers will turn to ordinary housing. On the contrary, *x* = 1 is the stable evolution point of developers; in other words, developers will choose developing green housing. The decision-making evolution phase diagrams of developers are shown in Figure 2.

#### 3.1.2. Consumers

The expected payoff *E*_21_ of consumers for purchasing green housing, *E*_22_ for purchasing ordinary housing, and their average expected payoff *E*_2_ are, respectively, as follows:

(4) $$\begin{array}{c} \left\{\begin{array}{c} E_{21}=xz\left[\left(S_{p}+\phi S_{Z1}+\eta S_{Z2}\right)-\left(A_{P}+A_{Z}\right)+\left(1-\theta \right)D_{2}\right]+x\left(1-z\right)\left[\left(S_{p}+\phi S_{Z1}+\eta S_{Z2}\right)-\left(A_{P}+A_{Z}\right)\right]\\ +\left(1-x\right)z\left[\left(S_{p}+\phi S_{Z1}+\eta S_{Z2}\right)-\left(A_{P}+A_{Z}\right)+\left(1-\theta \right)D_{2}\right]+\left(1-x\right)\left(1-z\right)\left[\left(S_{p}+\phi S_{Z1}+\eta S_{Z2}\right)-\left(A_{P}+A_{Z}\right)\right],\\ E_{22}=xz\left(S_{p}-A_{P}\right)+x\left(1-z\right)\left(S_{p}-A_{P}\right)+\left(1-x\right)z\left(S_{p}-A_{P}\right)+\left(1-x\right)\left(1-z\right)\left(S_{p}-A_{P}\right),\\ E_{2}=yE_{21}+\left(1-y\right)E_{22}. \end{array}\right) \end{array}$$

The replicator dynamics equation of consumers' decisions is as follows:

(5) $$\begin{array}{c} F\left(y\right)=\frac{\text{d}\left(y\right)}{\text{d}t}=y\left(E_{21}-E_{2}\right)=y\left(1-y\right)\left[z\left(1-\theta \right)D_{2}+\phi S_{Z1}+\eta S_{Z2}-A_{Z}\right]. \end{array}$$

The first derivatives with respect to *y* and the set *G* (*z*) are, respectively, as follows:

(6) $$\begin{array}{c} \frac{\text{d}\left(F\left(y\right)\right)}{\text{d}y}=\left(1-2y\right)\left[z\left(1-\theta \right)D_{2}+\phi S_{Z1}+\eta S_{Z2}-A_{Z}\right],\\ G\left(\text{z}\right)=\left[z\left(1-\theta \right)D_{2}+\phi S_{Z1}+\eta S_{Z2}-A_{Z}\right]. \end{array}$$

**Figure 3:**
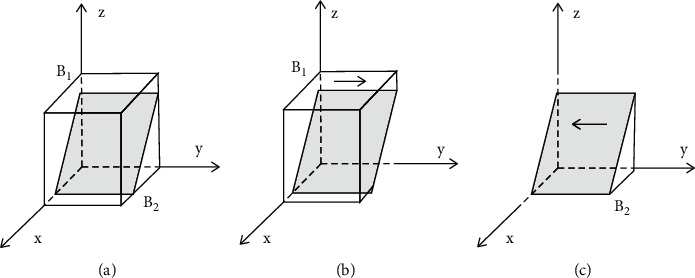
Evolution phase diagrams of consumers' decisions in the case of purchasing green housing. (a) *z*=*z*^*∗*^, *z* is equal to the stable point. (b) *z* > *z*^*∗*^, *z* is greater than the stable point. (c) *z* < *z*^*∗*^, *z* is less than the stable point.

Based on the stability principle of differential equations, the probability of consumers choosing purchasing green housing in a stable state must be satisfied by *F*(*y*)=0, where d(*F*(*y*))/d*y* < 0. Because ∂*G*(*z*)/∂*z* > 0, G(*z*) is the increasing function of *z*. Therefore, when *z*=(*A*_*Z*_ − *ϕS*_*Z*1_ − *ηS*_*Z*2_)/(1 − *θ*)*D*_2_=*z*^*∗*^, *G*(*y*)=0; at this point, d(*F*(*y*))/d*y* ≡ 0, *F*(*y*) ≡ 0, which means that in a group of consumers, any proportion of individuals choosing purchasing green housing is a stable strategy. When *z* < *z*^*∗*^, *G*(*z*) < 0, *F*′(*y*)*|*_*x*=0_ < 0, *F*′(*y*)*|*_*x*=1_ > 0; at this point, *y* = 0 is the stable evolution point of consumers, which means that when the probability of governments chooses providing incentive polices is less than (*A*_*Z*_ − *ϕS*_*Z*1_ − *ηS*_*Z*2_)/(1 − *θ*)*D*_2_, consumers will choose purchasing ordinary housing. On the contrary, *y* = 1 is the stable evolution point of consumers; in other words, consumers will choose purchasing green housing. The decision-making evolution phase diagrams of consumers are shown in Figure 3.

#### 3.1.3. Governments

The expected payoff *E*_31_ of governments choosing incentive polices, *E*_32_ of governments choosing no incentive polices, and their average expected payoff *E*_3_ are, respectively, as shown below:

(7) $$\begin{array}{c} \left\{\begin{array}{c} E_{31}=xy\left(G_{1}+G_{2}-D_{1}-D_{2}\right)+x\left(1-y\right)\left(G_{1}+G_{2}-D_{1}-\theta D_{2}-D_{3}\right)\\ +\left(1-x\right)y\left[G_{1}-D_{1}-\left(1-\theta \right)D_{2}\right]+\left(1-x\right)\left(1-y\right)\left(G_{1}-D_{1}\right),\\ E_{32}=xyG_{2}+x\left(1-y\right)\left(G_{2}-D_{3}\right),\\ E_{3}=zE_{31}+\left(1-z\right)E_{32}. \end{array}\right) \end{array}$$

The replicator dynamics equation of governments' decisions is as follows:

(8) $$\begin{array}{c} F\left(z\right)=\frac{\text{d}\left(z\right)}{\text{d}t}=z\left(E_{31}-E_{3}\right)=z\left(1-z\right)\left[G_{1}-D_{1}-x\theta D_{2}-y\left(1-\theta \right)D_{2}\right]. \end{array}$$

The first derivatives with respect to *z* and the set *G* (*x*) are, respectively, as shown below:

(9) $$\begin{array}{c} \frac{\text{d}\left(F\left(z\right)\right)}{\text{d}z}=\left(1-2z\right)\left[G_{1}-D_{1}-x\theta D_{2}-y\left(1-\theta \right)D_{2}\right],\\ G\left(x\right)=\left[G_{1}-D_{1}-x\theta D_{2}-y\left(1-\theta \right)D_{2}\right]. \end{array}$$

**Figure 4:**
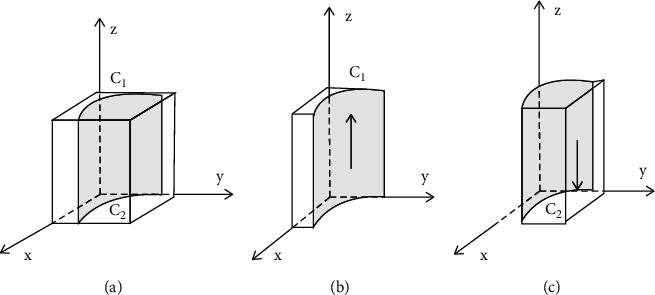
Evolution phase diagrams of governments in the case of choosing incentive polices. (a) *x*=*x*^*∗*^, *x* is equal to the stable point. (b) *x* < *x*^*∗*^, *x* is less than the stable point. (c) *x* > *x*^*∗*^, *x* is greater than the stable point.

Based on the stability principle of differential equations, the probability of governments choosing incentive polices in a stable state must be satisfied by *F*(*z*)=0, where d(*F*(*z*))/d*z* < 0. Because ∂*G*(*x*)/∂*y* < 0, *G*(*x*) is the decreasing function of *x*. Therefore, when *x*=[*G*_1_ − *D*_1_ − *y*(1 − *θ*)*D*_2_]/*θD*_2_=*x*^*∗*^, *G*(*x*)=0; at this point, d(*F*(*z*))/d*z* ≡ 0, *F*(*z*) ≡ 0, which means that in governments, any proportion of individuals choosing incentive polices is a stable strategy. When *x* < *x*^*∗*^, *G*(*x*) > 0, *F*′(*z*)*|*_*z*=0_ > 0, *F*′(*z*)*|*_*z*=1_ < 0; at this point, *z* = 1 is the stable evolution point of governments, which means that when the proportion of developers choosing developing green housing is less than [*G*_1_ − *D*_1_ − *y*(1 − *θ*)*D*_2_]/*θD*_2_, governments will provide incentive polices to encourage the development of green housing. On the contrary, *z* = 0 is the stable evolution point of governments; in other words, governments will not choose incentive polices. The decision-making evolution phase diagram of governments is shown in Figure 4.

### 3.2. Stability Analysis of Each Game Subject's Decision-Making

The simultaneous game of the replicator dynamics equations of three game subjects forms up a three-dimensional discrete dynamical system.

(10) $$\begin{array}{c} \left\{\begin{array}{c} F\left(x\right)=x\left(1-x\right)\left[y\left(2A_{P}+A_{Z}\right)+z\theta D_{2}+A_{Q}-A_{P}-C_{Z}\right]=0,\\ F\left(y\right)=y\left(1-y\right)\left[z\left(1-\theta \right)D_{2}+\phi S_{Z1}+\eta S_{Z2}-A_{Z}\right]=0,\\ F\left(z\right)=z\left(1-z\right)\left[G_{1}-D_{1}-x\theta {}_{2}^{D}-y\left(1-\theta \right)D_{2}\right]=0. \end{array}\right) \end{array}$$

Thirteen strategic equilibrium points are obtained by solving the equilibrium points of the discrete dynamical system: *E*_1_[0, 0, 0], *E*_2_[0, 0, 1], *E*_3_[1, 0, 0], *E*_4_[0, 1, 0], *E*_5_[1, 1, 0], *E*_6_[0, 1, 1], *E*_7_[1, 0, 1], *E*_8_[1, 1, 1], *E*_9_[0, *y*_0_, *z*_0_], *E*_10_[1, *y*′, *z*′], *E*_11_[*x*_1_, 0, *z*_1_], *E*_12_[*x*_2_, 1, *z*_2_], and *E*_13_[*x*_3_, *y*_1_, *z*_3_]. Among them, *E*_1_–E_8_ are the pure strategic equilibrium points for the three subjects of a green housing market; *E*_9_–*E*_12_ are the pure strategic equilibrium points for a single subject of a green housing market; and *E*_13_ is the mixed strategic equilibrium point. Some scholars have pointed out that the stable solution of the replicator dynamics system in a multigroup evolutionary game is a strict Nash equilibrium solution. Therefore, except for the eight equilibrium points (*E*_1_–*E*_8_), the remaining states are not asymptotically stable.

Based on the Lyapunov stability theory, the stability of a system at an equilibrium point is determined by the eigenvalues of a Jacobian matrix. When all the eigenvalues have negative real parts, an equilibrium point is asymptotically stable. When at least one of the eigenvalues has a positive real part, an equilibrium point is unstable. When all eigenvalues except those with zero real parts have negative real parts, an equilibrium point is in a critical state and the stability cannot be determined by the sign of an eigenvalue. Therefore, the Jacobian matrix of the discrete dynamical system can be obtained by integrating formula (10) as follows:

(11) $$\begin{array}{c} J=\left|\begin{array}{ccc} \frac{\partial F\left(x\right)}{\partial \left(x\right)} & \frac{\partial F\left(x\right)}{\partial \left(y\right)} & \frac{\partial F\left(x\right)}{\partial \left(z\right)}\\ \frac{\partial F\left(y\right)}{\partial \left(x\right)} & \frac{\partial F\left(y\right)}{\partial \left(y\right)} & \frac{\partial F\left(y\right)}{\partial \left(z\right)}\\ \frac{\partial F\left(z\right)}{\partial \left(x\right)} & \frac{\partial F\left(z\right)}{\partial \left(y\right)} & \frac{\partial F\left(z\right)}{\partial \left(z\right)} \end{array}\right|=\left|\begin{array}{ccc} J_{11} & x\left(1-x\right)\left(2A_{P}+A_{Z}\right) & x\left(1-x\right)\theta D_{2}\\ 0 & J_{22} & y\left(1-y\right)\left(1-\theta \right)D_{2}\\ z\left(z-1\right)\theta D_{2} & z\left(z-1\right)\left(1-\theta \right)D_{2} & J_{33} \end{array}\right|, \end{array}$$

where

(12) $$\begin{array}{c} J_{11}=\left(1-2x\right)\left[y\left(2A_{P}+A_{Z}\right)+z\theta D_{2}+A_{Q}-A_{P}-C_{Z}\right],\\ J_{22}=\left(1-2y\right)\left[z\left(1-\theta \right)D_{2}+\phi S_{Z1}+\eta S_{Z2}-A_{Z}\right],\\ J_{33}=\left(1-2z\right)\left[G_{1}-D_{1}-x\theta D_{2}-y\left(1-\theta \right)D_{2}\right]. \end{array}$$

*E*_1_–*E*_8_ were substituted into the Jacobian matrix to obtain their eigenvalues and analyze the stability of equilibrium points. The detailed results are shown in Table 2.

**Corollary 1 .**

When *A*_*Q*_ − *A*_*P*_ − *C*z < 0 and *G*_1_ − *d*_1_ < 0, the replicator dynamical system has a unique stable equilibrium point *E*_1_[0, 0, 0].

**Proof**

According to Chart 2, at this point, condition ① is met, *E*_1_[0, 0, 0] is the stable equilibrium point, and *C*_Z_ + *A_P_* − *A*_Q_ > 0, *D*_2_ + *D*_1_ − *G*_1_ > 0. Therefore, *E*_3_[1, 0, 0] and *E*_8_[1, 1, 1] are also instable points. It is concluded that *E*_1_[0, 0, 0] is unique.

Corollary 1 shows that when the potential payoff of developers developing green housing is less than the sum of incremental payoffs and incremental costs, and the payoff of governments promoting green housing is less than the sum of incremental payoffs and incremental costs, both developers and governments will not choose developing and promoting green housing. Consequently, a green housing market will disappear.

**Corollary 2 .**

When *A*_*Q*_ − *A*_*P*_ − *C*_*Z*_ > 0 and *G*_1_ − *D*_1_ − *θD*_2_ < 0, the replicator dynamical system has a unique stable equilibrium point *E*_3_[1, 0, 0].  Corollary 2 can be proved following the same way to proof Corollary 1.  Corollary 2 shows that when the potential payoff of developers developing green housing is greater than the sum of incremental payoffs and incremental costs, and the payoff of governments promoting green housing is less than the one without incentive policies, developers will still be willing to develop green housing. The reason is that developers think highly of huge potential payoffs in the future. However, this strategy is only theoretically stable. When consumers choose purchasing ordinary housing, a green housing market does not grow.

**Corollary 3 .**

When *D*_2_ + *D*_1_ − *G*_1_ < 0, the replicator dynamical system has a unique stable equilibrium point *E*_8_[1, 1, 1].  Corollary 3 can be proved following the same way to prove Corollary 1.  Corollary 3 shows that when the payoffs of incentive polices adopted by governments greater than their costs, developers will choose developing green housing, consumers will choose purchasing green housing, and governments will actively encourage them. The subjects of a green housing market jointly promote the sustainable and healthy growth of the market.

## 4. Numerical Simulation

In order to validate the stability of the evolution of green housing market subject behavior, the replicator dynamical system was assigned with numerical values for numerical simulation analysis. The determination method of numerical value is similar to the method of numerical selection in references [20–22], which is to obtain first-hand data by investigating consumers, developers, and leaders of government departments, and comparing the dimensions of the data to obtain the relative value [26]. Due to the limited length, this study selected numerical values, where *A*_*P*_ = 50, *A*_*Z*_ = 40, *θ* = 0.5, *D*_2_ = 40, *A*_*Q*_ = 30, *C*_*Z*_ = 50, *φ* = 0.5, *S*_*Z*1_ = 30, *η* = 0.5, *S*_*Z*2_ = 30, *G*_1_ = 160, and *D*_1_ = 90. The impacts of *A*_*Z*_, *A*_*Q*,_*θ*, *η,* and *D*_2_ on the process and results of the system evolutionary game were analyzed when the conditions of *C*_*Z*_ − *A*_*Z*_ − *A*_*P*_ − *θD*_2_ − *A*_*Q*_ < 0, *A*_*Z*_ − *φS*_*Z*1_ − *ηS*_*Z*2_ − (1 − *θ*) *D*_2_ < 0, and *D*_2_ + D_1_ − *G*_1_ < 0 were satisfied.

### 4.1. Impacts of Incremental and Potential Payoffs of Developers Choosing Developing Green Housing on the System Evolutionary Game

**Figure 5:**
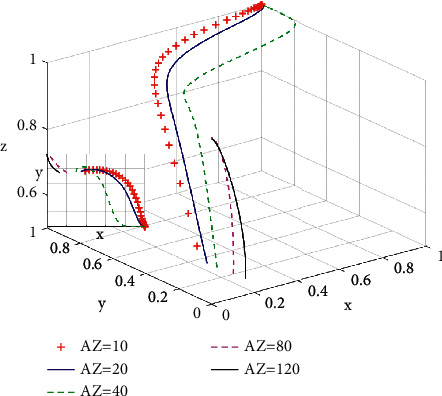
Influence of incremental income *A*_*Z*_ on the system evolutionary game.

According to Figure 5, when a green housing market is approaching a state of stability, the incremental payoff of developing green housing for developers is increasing, and the evolutionary speed of green housing development is accelerating. With the increase of *A*_*Z*_, the probability of developing green housing for developers is increasing. When *A*_*Z*_ reaches over 80, however, the system enters a state of instability. At this time, the increase of green housing incremental payoffs brings the increase of incremental costs for consumers purchasing green housing. When incremental costs reach a certain extent, consumers turn to ordinary housing, leading to a state of instability for a green housing market. This shows as a profitable enterprise, the developers excessively pass on the cost of green housing to consumers, thereby result in green housing market instability, which is in accord with Jiang and Payne [27].

**Figure 6:**
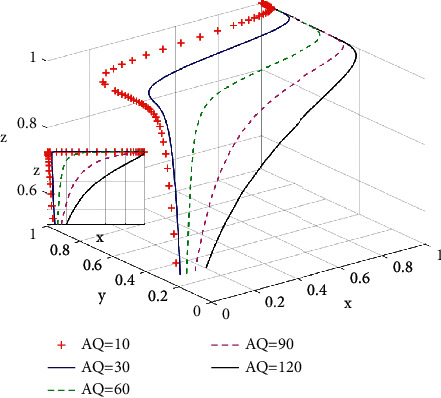
Influence of potential returns *A*_*Q*_ on the system evolutionary game.

As can be seen from Figure 6, when a green housing market is approaching a state of stability, the increase of potential income of green housing obtained by developers will accelerate the evolution speed of green housing development by developers. The application of green strategies such as the solar system and water saving technology can help contribute to building up developers' competitive advantages and bring a potential income for developers [28]. In other words, with the increase of *A*_*Q*_, the probability of developing green housing for developers is increasing. In the case where governments provide incentive polices, the greater potential payoffs are the earlier a green housing market enters a stable state.

### 4.2. Impacts of Consumers' Green Housing Subsidies and Green Perception on the System Evolutionary Game

**Figure 7:**
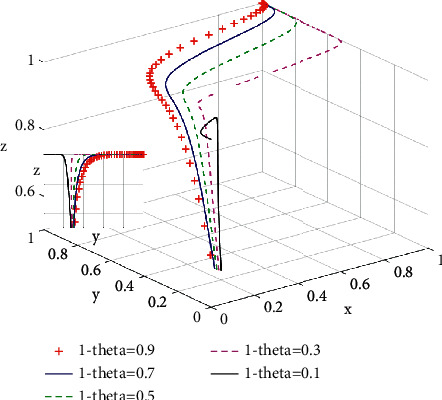
Influence of incremental return coefficient 1 − *θ* on the system evolutionary game.

According to Figure 7, when the total subsidy *D*_2_ for green housing remains unchanged and 1 − *θ* does not exceed 0.1 (i.e., the proportion of subsidies for consumers is low), the proportion of consumers choosing purchasing green housing drops from 0.3 to 0. However, when the proportion of subsidies 1 − *θ* increases gradually, the proportion of consumers choosing purchasing green housing increases, and the evolutionary speed of consumers choosing green housing increases as well. He et al. [29] support the idea that the government encourages potential buyers of green houses to purchase through financial subsidies.

**Figure 8:**
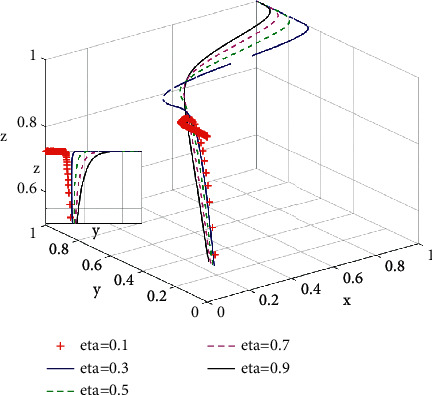
Influence of green perception coefficient *η* on the system evolution game.

Green perception refers to consumers' satisfaction and comfort brought by green housing and essentially represents consumers' cognitive level of environmental protection, thereby determines the willingness to purchase, which is consistent with Guo et al. [30]. As shown in Figure 8, when green perception is low (i.e., *η* is less than 0.1), even if subsidies from governments for purchasing green housing are in place, the proportion of consumers choosing purchasing green housing drops from 0.3 to 0 at the beginning of system evolution. This means consumers only purchase ordinary housing. However, with the increase of green perception *η*, the proportion of consumers choosing purchasing green housing increases and the evolutionary speed of consumers choosing green housing increases as well.

### 4.3. Impacts of the Total Amount of Subsidies and Social Promotion for Green Housing on the System Evolutionary Game

**Figure 9:**
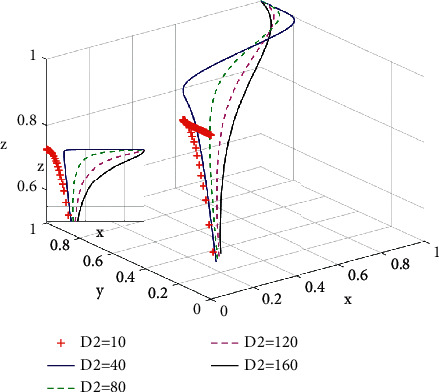
Influence of total green housing subsidy *D*_2_ on the system evolution game.

As shown in Figure 9, when the incremental payoff *θ* is constant, the total amount of subsidies *D*_2_ provided by governments as incentive polices has a significant impact on developers' decisions. When the total amount of subsidies *D*_2_ is less than 10, the proportion of developers choosing developing green housing decreases from 0.3 to 0. When the total amount of subsidies *D*_2_ is gradually increasing, the proportion of developers choosing developing green housing is gradually increasing from the initial value 0.3, and the evolutionary speed of developers choosing developing green housing is increasing as well, which is in line with the studies by He and Chen [16].

**Figure 10:**
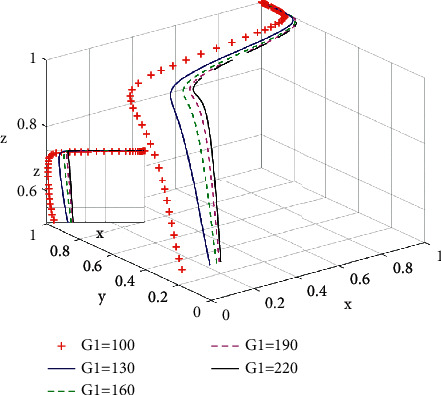
Influence of social promotion effect *G*_1_ on the systematic evolutionary game.

According to Figure 10, when governments use propaganda of environmental protection and deploy regulation and monitoring on environment, the social promotion effect of green housing is limited. In other words, when *G*_1_ = 100, the proportion of developers choosing green housing gradually decreases from the initial value 0.3. When intensive propaganda of environmental protection and strong environmental monitoring and regulation are in place, the social promotion of green housing becomes effective. With the increase of the proportion of government incentive policies, the proportion of developers choosing green housing increases and the evolutionary speed of the system increases.

## 5. Conclusion and Implications

The purpose of this research was to examine that how the behavioral decisions of developers, consumers, and governments among green housing market subjects affect the growth of green housing. First, this study builds a tripartite evolutionary game model to investigate a green housing marketing, which includes developers, consumers, and governments. Second, this study obtains a three-dimensional discrete dynamic system and identifies the stable conditions of strategy portfolios to reach the equilibrium points of the game system based on the Lyapunov stability theory. Finally, this study assigns real-world values to the proposed tripartite evolutionary game model and conducts numerical simulation to test the influence and relationship of each factor on system evolution, validating the proposed model. Moreover, the results of this study reveal that developers should not blindly pursue the incremental payoffs brought by green housing; otherwise, consumers may turn to ordinary housing. Similarly, the potential earnings from green housing will make green housing more favorable to developers. Moreover, regarding consumers, the perception of green housing determines the demand intensity from consumers. Meanwhile, the more subsidies provided by governments, the more enthusiastic consumers will be in purchasing green housing. Furthermore, when governments do not provide or provide mild subsidies, both developers and consumers will embrace ordinary housing. In addition, governments can effectively increase the initiatives of developers and improve the awareness of consumers toward green housing through intensive propaganda of greenness and environmental protection and strong regulation of environment.

In addition, this study generates the following implications to the sustainable and healthy growth of green housing markets in China.

First, developers are the suppliers in a green housing market, who undertake the development task of green housing. In the process of green housing development, it is not feasible for developers to blindly pursue incremental payoffs brought by green housing. The increase of incremental payoffs for developers will result in the increase of incremental costs for consumers, where consumers may turn to ordinary housing as they cannot afford green housing. This is evident in our simulation when *A*_*Z*_ climbs over 80, the system of a green housing market enters a state of instability. Similarly, the increase of potential payoffs for developers will promote the development of green housing. There are various approaches to increase the potential payoffs for developers, such as taxation deductions and mortgage discounts. By applying these approaches, more developers will be encouraged to enter a green housing market.

Second, consumers are the demand side in a green housing market. Their perceptions toward green housing determines their demand intensity. Therefore, strengthening the publicity of green housing, improving consumers' understanding of green housing, and enhancing consumers' awareness of green housing are necessary measures to initiate the motivation of consumers to purchase green housing. At the same time, subsidies provided by governments to purchase green housing have a strong effect on the will of consumers to purchase green housing. This is demonstrated in our simulation where the increase of the subsidy 1 − *θ* for consumers will lead to the increase of the proportions of consumers who purchase green housing. Therefore, high subsidies can boost the demand side in a green housing market, facilitating the healthy and sustainable growth of green housing markets in China.

Lastly, governments are the facilitators and regulators in a green housing market. Governments formulate and implement relevant incentive polices to promote the growth of a green housing market. The incentives and subsidies provided by governments play a key role in cultivating and promoting a green housing market. If governments do not provide or provide little subsidies, both developers and consumers will turn to ordinary housing due to the large incremental costs of developing and purchasing green housing. Our simulation generates a similar result. When the total amount of the subsidy for green housing is less than ten units, developers will choose developing ordinary housing. In addition to subsidies, governments have other measures to stimulate the growth of a green housing market, such as intensive propaganda of environmental protection and strong regulations on built environment. By doing this, governments can boost the initiative of developers to develop green housing and the understanding of green housing for consumers. This is supported in our simulation where a higher effect of green housing promotion can result in a higher supply and demand of green housing. Therefore, intense incentives can accelerate the growth of green housing markets in China. While this study has two limitations such as considering merely the mainland Chinese greenhouse market, ignoring the difference of consumers' educational background and capital, which need to be further researched in the future.

## Figures and Tables

**Table 1:** Mixed strategy payoff matrix of developers, consumers, and governments.

The game party		Consumers^②^	Governments^③^	
			Providing incentive polices *z*	Providing no incentive polices(1 − *z*)
Developers^①^	Developing green housing *x*	Purchasing green housing *y*	①: *A*_*P*_ + *A*_*Z*_ − (*C*_*P*_ + *C*_z_) + *θD*_2_ + *A*_*Q*_	①: *A*_*P*_ + *A*_*Z*_ − (*C*_*P*_ + *C*_*z*_)+*A*_*Q*_
			②: *S*_*P*_ + *φS*_*Z*1_ + *ηS*_*Z*2_ − *A*_*P*_ − *A*_*Z*_ + (1 − *θ*)*D*_2_	②: *S*_*P*_ + *φS*_*Z*1_ + *ηS*_*Z*2_ − *A*_*P*_ − *A*_*Z*_
			③: *G*_1_ + *G*_2_ − *D*_1_ − *D*_2_	③: *G*_2_
		Purchasing ordinary housing (1 − *y*)	①: –(*C*_*P*_ + *C*_z_) + *θD*_2_ + *A*_*Q*_	①: –(*C*_P_ + *C*_z_) + *A*_*Q*_
			②: *S*_*P*_ − *A*_*P*_	②: *S*_*P*_ − *A*_*P*_
			③: *G*_1_ + *G*_2_ − *D*_1_ − *θD*_2_ − *D*_3_	③: *G*_2_ − *D*_3_
	Developing ordinary housing (1 − *x*)	Purchasing green housing *y*	①: –*C*_*P*_	①: –*C*_*P*_
			②: *S*_*P*_ + *φS*_*Z*1_ + *ηS*_*Z*2_ − *A*_*P*_ − *A*_*Z*_ + (1 − *θ*)*D*_2_;	②: *S*_*P*_ + *φS*_*Z*1_ + *ηS*_*Z*2_ − *A*_*P*_ − *A*_*Z*_
			③: *G*_1_ − *D*_1_ − (1 − *θ*)*D*_2_	③: 0
		Purchasing ordinary housing (1 − *y*)	①: *A*_*P*_ − *C*_*P*_	①: *A*_*P*_ − *C*_*P*_
			②: *S*_*P*_ − *A*_*P*_	②: *S*_*P*_ − *A*_*P*_
			③: *G*_1_ − *D*_1_	③: 0

**Table 2:** Eigenvalues of the Jacobian matrix and local stability judgment of equilibrium point.

Equant equation	Eigenvalues of the Jacobian matrix		Stability conclusion	Condition
	*λ*_1_, *λ*_2_, *λ*_3_	Real component symbol		
*E*_1_[0, 0, 0]	*A*_*Q*_ − *A*_*P*_ − *C*_*Z*_, *φS*_*Z*1_ + *ηS*_*Z*2_ − *A*_Z_, *G*_1_ − *D*_1_	(×, −, ×)	Stable point	①
*E*_2_[0, 0, 1]	*θD*_2_ + *A*_*Q*_ − *A*_*P*_ − *C*_*Z*_, (1 − *θ*)*D*_2_ + *φS*_*Z*1_ + *ηS*_*Z*2_ − *A*_*Z*_, *D*_1_ − *G*_1_	(×, +, ×)	Instable point	—
*E*_3_[1, 0, 0]	*C*_*Z*_ + *A*_*P*_ − *A*_*Q*_, *φS*_*Z*1_ + *ηS*_*Z*2_ − *A*_*Z*_, *G*_1_ − *D*_1_ − *θD*_2_	(×, −, ×)	Stable point	②
*E*_4_[0, 1, 0]	*A*_*Q*_ + *A*_*P*_ + *A*_*Z*_ − *C*_*Z*_, *A*_*Z*_ − *φS*_*Z*1_ − *ηS*_*Z*2_, *G*_1_ − *D*_1_ − (1 − *θ*)*D*_2_	(+, +, −)	Instable point	—
*E*_5_[1, 1, 0]	*C*_*Z*_ − *A*_*P*_ − *A*_*Z*_ − *A*_*Q*_, *A*_*Z*_ − *φS*_*Z*1_ − *ηS*_*Z*2_, *G*_1_ − *D*_1_ − *D*_2_	(−, −, +)	Instable point	—
*E*_6_[0, 1, 1]	*θD*_2_+*A*_*Q*_ + *A*_*P*_ + *A*_*Z*_ − *C*_*Z*_, *A*_*Z*_ − *φS*_*Z*1_ − *ηS*_*Z*2_ − (1 − *θ*)*D*_2_, (1 − *θ*)*D*_2_ + *D*_1_ − *G*_1_	(+, −, −)	Instable point	—
*E*_7_[1, 0, 1]	*C*_*Z*_ + *A*_*P*_ − *θD*_2_ − *A*_*Q*_, (1 − *θ*)*D*_1_ + *φS*_*Z*1_ + *ηS*_*Z*2_ − *A*_*Z*_, *θD*_2_+*D*_1_ − *G*_1_	(+, +, −)	Instable point	—
*E*_8_[1, 1, 1]	*C*_*Z*_ − *A*_*Z*_ − *A*_*P*_ − *θD*_2_ − *A*_*Q*_, *A*_*Z*_ − *φS*_*Z*1_ − *ηS*_*Z*2_ − (1 − *θ*)*D*_2_, *D*_2_ + *D*_1_ − *G*_1_	(−, −, ×)	Stable point	③

*Note. x* means the symbol is uncertain, ① *A*_*Q*_ − *A*_*P*_ − *C*_*Z*_ < 0, *G*_1_ − *D*_1_ < 0; ② *C*_*Z*_ + *A*_*P*_ − *A*_*Q*_ < 0, *G*_1_ − *D*_1_ − *θ*D_2_ < 0; ③ *D*_2_ + *D*_1_ − *G*_1_ < 0.

## Data Availability

The simulation part of this paper involves data, which are relative data and represent the degree of correlation. The data come from two parts: one is based on the research data of previous scholars and the other is obtained through interviews with relevant personnel of developers, consumers, and governments involved in the real estate market.
